# Human Transcriptome Array Analysis Identifies CDR2 as a Novel Suppressed Gene for Kawasaki Disease

**DOI:** 10.3390/diagnostics12020240

**Published:** 2022-01-19

**Authors:** Ying-Hsien Huang, Kuang-Den Chen, Kuang-Che Kuo, Mindy Ming-Huey Guo, Ling-Sai Chang, Ya-Ling Yang, Ho-Chang Kuo

**Affiliations:** 1 Kawasaki Disease Center, Kaohsiung Chang Gung Memorial Hospital and Chang Gung University College of Medicine, Kaohsiung 83301, Taiwan; yhhuang123@yahoo.com.tw (Y.-H.H.); dennis8857@gmail.com (K.-D.C.); light@cgmh.org.tw (K.-C.K.); mindymhguo@yahoo.com.tw (M.M.-H.G.); joycejohnsyoko@gmail.com (L.-S.C.); 2 Department of Pediatrics, Kaohsiung Chang Gung Memorial Hospital and Chang Gung University College of Medicine, Kaohsiung 83301, Taiwan; 3 Institute for Translational Research in Biomedicine, Liver Transplantation Center and Department of Surgery, Kaohsiung Chang Gung Memorial Hospital and Chang Gung University College of Medicine, Kaohsiung 83301, Taiwan; 4 Department of Anesthesiology, Kaohsiung Chang Gung Memorial Hospital and Chang Gung University College of Medicine, Kaohsiung 83301, Taiwan; inr453@cgmh.org.tw

**Keywords:** CDR2, Kawasaki disease, suppressed gene

## Abstract

Kawasaki disease (KD) is a febrile childhood vasculitis that involves the coronary arteries. Most previous studies have focused on the genes activated in the acute phase of KD. However, in this study, we focused on suppressed genes in the acute stage of KD and identified novel targets with clinical significance and potential prognostic value for KD patients. We enrolled 18 patients with KD, 18 healthy controls (HC), and 18 febrile controls (FC) for human transcriptome array analysis. Another 19 healthy controls, 20 febrile controls, and 31 patients with KD were recruited for RT-PCR validation of target mRNA expressions. The results of Human Transcriptome Array (HTA) 2.0 showed 461 genes that were significantly higher in KD and then normalized after IVIG, as well as 99 suppressed genes in KD. Furthermore, we identified the four genes in KD with the most downregulation, including BCL11B, DUSP2, DDX24, and CDR2, as well as the upregulation of their expression following IVIG administration. The mRNA expression of CDR2 by qRT-PCR was the most compatible with the pattern of the HTA2.0 results. Furthermore, we found higher DDX24 mRNA expression in KD patients with CAL when compared to those without CAL 3 weeks after IVIG administration. In summary, activated gene expression represented a majority in the immune response of KD. In this study, we identified CDR2 as a novel suppressed gene for Kawasaki disease via human transcriptome array analysis and DDX24 associated with CAL formation, which may contribute to further understanding of CAL pathogenesis in KD.

## 1. Introduction

Kawasaki disease (KD) is the most common cause of acquired heart disease in children, especially in Asian countries such as Japan and Taiwan [1,2,3]. The main features of classical KD include a fever lasting for at least 5 days and at least four out of the five of the following symptoms: erythema of the oral mucosa (fissured lips, strawberry tongue), bilateral non-suppurative conjunctivitis, lymphadenopathy, edema or erythema of the hands or feet, and a polymorphous rash [4]. KD mostly involves inflammation of medium-sized vessels and can result in multiple cardiovascular sequelae, most commonly coronary artery aneurysms (CAA), as well as myocardial ischemia or infarction later in life [5]. In the 1980s, intravenous immunoglobulin (IVIG) was proven to be the most effective treatment for KD, and most children with KD demonstrate rapid resolution of fever and symptoms following the administration of IVIG [6,7]. We have also observed an increased incidence of KD in recent years [3,8].

In our previous research using Human Transcriptome Array 2.0 (HTA 2.0, Affymetrix, Santa Clara), we found that patients with KD exhibited the most increased inflammatory gene expression in the acute phase of KD compared to controls; meanwhile, these genes decrease after patients receive intravenous immunoglobulin (IVIG) therapy [9,10,11,12]. Among them, KD patients showed significantly increased expression of almost all known toll-like receptors (TLRs), with the exception of TLR3 and TLR7 [9]. TLRs are membrane receptors that aid the innate immune system in recognizing both pathogen-associated molecular patterns that originate from infectious pathogens, and damage-associated molecular patterns released by dying cells [13]. Of particular interest, we also demonstrated that the expression of CD 177 genes of leukocytes play an important role in the pathogenesis and primary IVIG response during the acute inflammatory process of KD [10]. In general, these results support the hypothesis that KD is a disease of immune overactivation [14]. 

Although the exact pathogen or trigger of KD remains unclear and varied, a T-helper (Th)1/Th2 imbalance has been found to contribute to the development of KD [14]. Guo et al. demonstrated the upregulation of T helper (Th) 17 cells and the downregulation of Treg cells in the acute stage of KD [15]. Growing evidence has revealed several possible mechanisms by which IVIG might induce such a rapid resolution of inflammation in KD [16]. Antibodies in IVIG may neutralize the antigen or immune complex that modulates multiple NK cell and T-cell functions of KD [17] and affects Treg activation via the secretion of such anti-inflammatory cytokines as Treg-related IL-10 and FoxP3 [15,16,17]. Genome-wide association studies have revealed a functional single nucleotide polymorphism (SNP) in the ITPKC gene, encoding inositol 1,4,5-trisphosphate 3-kinase C, whose role is to negatively regulate T-cell activation through the calcium signaling pathway, which is associated with susceptibility to KD [18,19] and coronary artery aneurysms [20].

Most previous studies have focused on the genes activated in the acute phase of KD [10,11]. In this study, we focused on the suppressed genes in the acute stage of KD and identified novel targets with a clinical significance and potential prognostic value for KD patients.

## 2. Methods

### 2.1. Subject Recruitment

We enrolled 18 patients with KD, 18 healthy controls (HC), and 18 febrile controls (FC) for human transcriptome array analysis. A second cohort of 9 healthy controls, 10 febrile controls, and 14 patients with KD were recruited to further confirm target mRNA expression and detect serum mitochondrial DNA. All KD patients for this study met the criteria set forth by the American Heart Association [4]. All patients received at least one dose of IVIG, 2 g/kg/dose infused over 12 h according to current practice guidelines [4]. IVIG resistance is defined as persistent fever 48 h after completion of IVIG infusion, and patients with IVIG resistance were given a second dose of IVIG (2 g/kg/dose). Blood samples were obtained from KD patients within 24 h prior to IVIG therapy and then at least 21 days after IVIG therapy. We defined the presence of coronary artery lesions (CAL) as the internal diameter of the coronary arteries having a z-score of ≥2.5 or as the absolute measurement of the coronary arteries being ≥3 mm (if younger than 5 years old) or ≥4 mm (if older than 5 years old) [21,22]. Informed consent was obtained from the parents or guardians of all patients included in this study. This study was approved by the Internal Review Board (#202001350A3) of our hospital. 

### 2.2. Human Transcriptome Array 

As previous reports have described [23], total mRNA samples from 18 healthy controls, 18 febrile controls, and 18 KD patients 24 h before IVIG was given (KD1 group), and 18 KD patients 21 days after IVIG therapy (KD2 group) were pooled together into three RNA libraries, each containing RNA samples from 6 patients. All RNA samples were then prepared for hybridization to the GeneChip^®^ Human Transcriptome Array 2.0 (HTA 2.0, Affymetrix, Santa Clara) using the WT PLUS Reagent kit. Hybridized HTA 2.0 microarray chips were checked for quality, and the gene expression data was then analyzed with commercially available software (Partek, St. Louis, MI, USA).

### 2.3. mRNA Expression with Quantitative Real Time Reverse Transcription Polymerase Chain Reaction

We confirmed leukocyte mRNA expression results of B-cell leukemia/lymphoma 11B (BCL11B), cerebellar degeneration-related protein 2 (CDR2), DEAD (Asp-Glu-Ala-Asp) box RNA helicase 24 (DDX24), and dual specificity phosphatase 2 (DUSP2) using qRT-PCR. Total RNA obtained from leukocytes was first transformed into cDNA by following the manufacturer’s instructions (cDNA High-Capacity cDNA Reverse Transcription kit, Applied Biosystems, Cat. 4368813). Quantitative RT-PCR was performed on the LightCycler R480 RT-PCR System (Roche Molecular System, Pleasanton, CA, USA) by adding 2.5 ng/μL of cDNA from each sample with 0.2 μL (10 μM) of forward and reverse primers and 5 L of SYBR Green Master Mix (ABI, Cat. No. 4367659). We calculated relative mRNA expression levels by comparing the RT-PCR cycle number required to reach target fluorescence (the comparative threshold cycle method) using the equation 2^−(^^ΔCTtarget−^^ΔCTcalibrator)^ (i.e., 2^−^^ΔΔCT^). We conducted a melting curve analysis to confirm a single, specific amplification product and all experiments were performed twice to verify amplification efficiency. Primers included in this study included: 18S: (F) GTAACCCGTTGAACCCCATT, (R) CCATC CAATCGGTAGTAGCG; DUSP2: (F) CCTGTGGAGGACAACCAGATGG, (R) TG CCAGACAGATGGTGGCAGAG; DDX24: (F) AGGCCGGAGCTGAGACTAGAT C, (R) GTCTGAGACAGTGCCTCCAGTC; BCL11B: (F) CACCTGCTCTCACCCA CGAAAG, (R) GGCACGCAGAGGTGAAGTGATC; CDR2: (F) TACTAATTTGCC TATTGCCTATCG, (R) ATGGAAGTGGATCAGAGAAC.

### 2.4. Statistical Analysis

All values are expressed as mean ± standard error (SE). Once chips passed the quality control criteria, we evaluated them using Partek (Partek, St. Louis), a commercial software specifically designed to analyze microarray data, as previously described [10]. We used the Shapiro–Wilk test for data normality and the Kruskal–Wallis test for significance between multiple group comparison. Quantitative data were analyzed using a paired *t*-test or Mann–Whitney U test for two-group comparison when appropriate. Two-sided *p*-values less than 0.05 were considered statistically significant. Statistical analysis was performed using SPSS version 14.0 (SPSS, Inc., Chicago, IL, USA). 

## 3. Results

### 3.1. Demographic Data

The demographic data of the 18 KD patients and 18 healthy and 18 febrile controls included for HTA 2.0 microarray analysis have been previously published elsewhere [9]. Full microarray data have been uploaded and can be accessed on the NCBI GEO database (Series GSE109351). We also recruited an additional cohort of 19 healthy controls, 20 febrile controls, and 31 patients with KD in a case–control study (Table 1). We observed no significant differences in terms of gender between the three groups. However, patients with KD were significantly younger than both the healthy and febrile controls (3.2 ± 0.4, 3.5 ± 0.5, 1.7 ± 0.2 years of age, respectively, *p* < 0.001). On average, prior to IVIG therapy, patients with KD had higher white blood cell counts (9.3 ± 0.5, 7.5 ± 0.9, 11.4 ± 0.6, 1000 cells/uL, *p* < 0.001, respectively) and lower hemoglobulin levels (12.5 ± 0.2, 12.2 ± 0.2, 11.3 ± 0.2 mg/L, *p* < 0.001, respectively) than both healthy and febrile controls. Of the 31 patients with KD, 14 patients (45.2%) had evidence of CAL and one patient had IVIG resistance (3.3%; Table 1). 

### 3.2. Patients with Acute KD Have Less Suppressed Than Increased Genes Expression Compared to Controls Using HTA 2.0 

**Figure 1:**
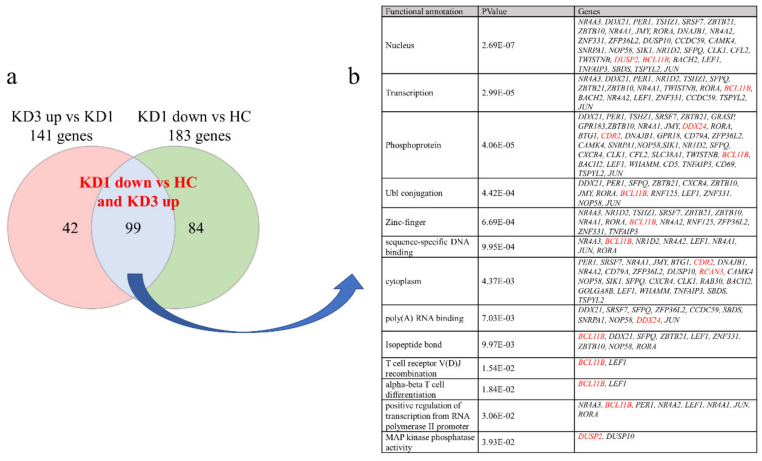
Patients with acute-stage KD have less suppressed than increased genes compared to controls as determined by HTA 2.0. (**a**) Putative differentiated gene targets between Kawasaki disease (KD) and controls were screened by Targetscan7.2. A Venn diagram illustrates numbers between groups; (**b**) A KEGG pathway analysis of 99 putative target genes in humans. In addition, we observed that BCL11B, DUSP2, DDX24, and CDR2 demonstrated the most significant fold-changes between KD patients (KD1) before IVIG and 3 weeks after IVIG administration (KD3). Note that BCL11B, DUSP2, DDX24, and CDR2 (highlighted in red) are present in all the pathways.

**Figure 2:**
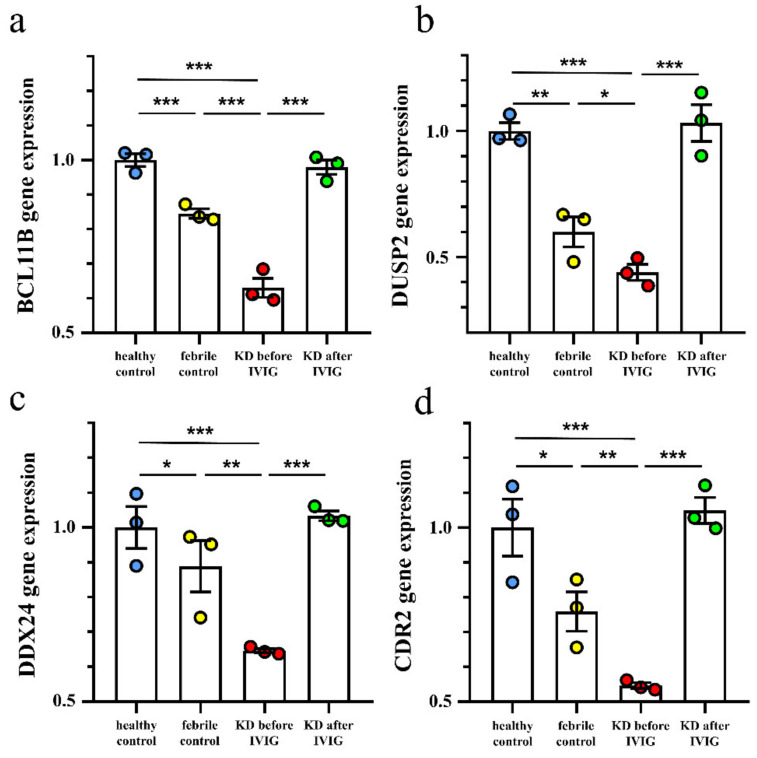
Comparison of BCL11B (**a**), DUSP2 (**b**), DDX24 (**c**), and CDR2 (**d**) expression determined by GeneChip^®^ Human Transcriptome Array 2.0 between acute-stage Kawasaki disease patients and control subjects. * Indicates significance (*p*< 0.05), ** indicates significance (*p* < 0.01). *** indicates significance (*p* < 0.001). Data are expressed as mean ± standard error for the three replications.

In this study, we focused on the variations in genetic expression between control subjects and KD patients of different stages. In the beginning, according to the HTA 2.0 results, a total of 461 significantly higher genes and 99 lower genes, respectively, were shown in KD prior to IVIG administration compared to controls, which were normalized after IVIG (Figure 1a). We focused on the suppressed genes in the acute stage of KD, so the 99 suppressed genes were further subjected to the DAVID Bioinformatics Resource 6.8 (https://david.ncifcrf.gov/tools.jsp (accessed on 25 November 2021) (2021 Update) to identify the Kyoto Encyclopedia of Genes and Genomes (KEGG) human pathways involved, as shown in Figure 1b. In addition, we observed that BCL11B, DUSP2, DDX24, and CDR2 demonstrated the most significant fold-change between KD patients before and after IVIG administration. As shown in Figure 2, the mRNA levels of BCL11B, DUSP2, DDX24, and CDR2 were significantly lower in KD patients than in the healthy control and febrile control groups. These BCL11B, DUSP2, DDX24, and CDR2 values significantly increased in KD patients after undergoing IVIG treatment. We also noted that they were present in all 13 KEGG pathways (Figure 1b).

### 3.3. Validation of These Suppressed Genes Expression in KD Patients by qRT-PCR 

**Figure 3:**
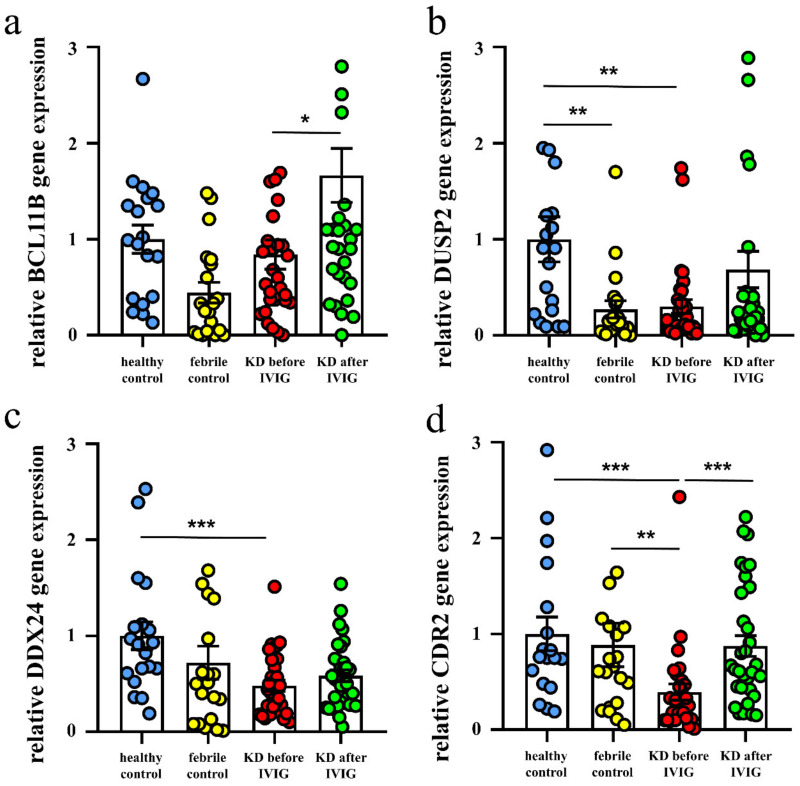
Comparison of BCL11B (**a**), DUSP2 (**b**), DDX24 (**c**), and CDR2 (**d**) expression determined by quantitative real-time reverse transcription polymerase chain reaction between acute-stage Kawasaki disease patients and control subjects. * Indicates significance (*p* < 0.05), ** indicates significance (*p* < 0.01). *** indicates significance (*p* < 0.001) Data are expressed as mean ± standard error for the three replications.

**Figure 4:**
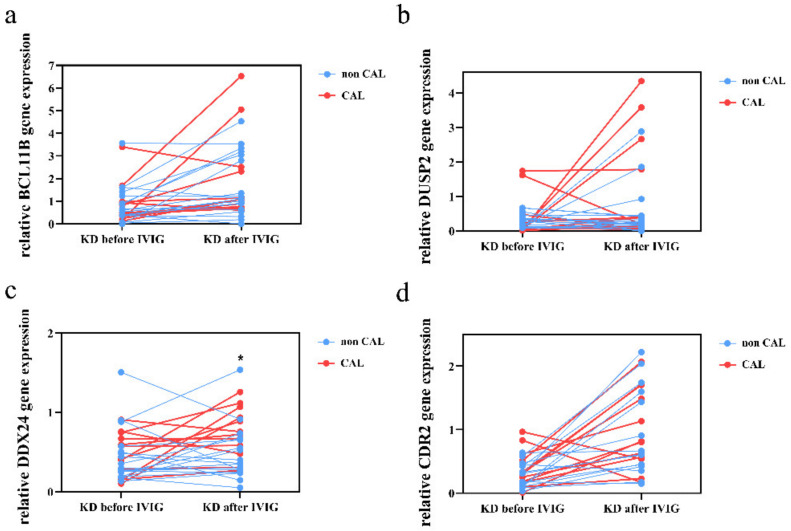
Comparison of BCL11B (**a**), DUSP2 (**b**), DDX24 (**c**), and CDR2 (**d**) expression determined by quantitative real-time reverse transcription polymerase chain reaction between Kawasaki disease patients with or without coronary arterial lesion (CAL). * Indicates significance (*p* < 0.05) Data are expressed as mean ± standard error for the three replications.

We then conducted qPCR assays on BCL11B, DUSP2, DDX24, and CDR2 to investigate the mRNA levels in a separate cohort of 31 KD patients, 20 febrile controls, and 19 healthy controls. As shown in Figure 3, our results showed decreased DUSP2, DDX24, and CDR2 in KD patients compared to the healthy or febrile controls. In addition, the mRNA of BCL11B and CDR2 considerably increased 3 weeks after IVIG administration (Figure 3). Notably, the mRNA expression of CDR2 by qRT-PCR is most compatible with the pattern of the HTA2.0 results. We also found higher DDX24 mRNA expression in KD patients with CAL compared to those without CAL 3 weeks after IVIG administration (*p* = 0.031; Figure 4). The BCL11B, DUSP2, and CDR2 mRNA expressions are not associated with CAL development. 

## 4. Discussion

KD is a systemic inflammatory disease, and our HTA 2.0 results showed a total of 461 genes that were significantly higher in KD prior to IVIG administration than in controls, which then normalized after IVIG. Of the 99 suppressed genes in KD, our noteworthy observations identified four genes with the most downregulation: BCL11B, DUSP2, DDX24, and CDR2 in KD, as well as their upregulation of expression after IVIG administration. In another independent cohort, the mRNA expression of CDR2 by qRT-PCR was most compatible with the pattern of the HTA2.0 results. Furthermore, we found higher DDX24 mRNA expression in KD patients with CAL compared to those without CAL 3 weeks after IVIG administration.

Our previous study indicated that the pyroptosis of leukocytes that focused on neutrophils or monocytes played a role in the pathogenesis of KD. Neutrophils recruited in large numbers to these disease-associated tissues or trapped in areas of microcirculatory impairment are exposed to profound levels of hypoxia [24,25]. With the stimulation of neutrophil extracellular traps (NETs), the expression of vascular endothelial growth factor A (VEGF-A) and hypoxia-inducible factor-1α (HIF-1α) increased, both of which were related to the pathological mechanisms of KD [26,27]. HIF-1α produced by macrophages and neutrophils is involved in the regulation of the inflammatory response and the intensification of the innate immune response [28]. P300 has previously been identified as a co-activator of HIF1α (hypoxia-inducible factor 1 alpha) in HIF-1 signaling pathways [29]. In addition to being a consequence of inflammation, hypoxia can drive inflammatory processes; increased vascular leak and endothelial damage and impaired resolution of infection have been demonstrated in hypoxic mice and humans [30]. CDR2 is expressed in the central nervous system, and its ectopic expression in tumor cells of patients with gynecological malignancies is associated with paraneoplastic cerebellar degeneration [31,32]. Additionally, CDR2 can be detected in vascular smooth muscle cells of rats and humans [33]. Interestingly, CD8+cytotoxic T cells will fail to activate T cells expressing CDR2 in response to epithelial cells expressing CDR2 [34]. Balamurugan et al. indicated that the overexpression of the CDR2 gene plays an important role in repressing HIF-1 transactivation activity by interfering with p300 recruitment in their study on solid tumors [35]. Buenafe et al. also showed that T-cell receptor CDR2 peptide immunotherapy was effective in regulating pathogenic T-cells via the activity of the Foxp3(+) regulatory T-cells [33], which is consistent with our previous findings that IVIG therapy for KD results in increased expression of Treg-related FoxP3 [17]. Herein, we showed that the suppression of the CDR2 gene of leukocytes and its increase after IVIG administration may play a role in the pathogenesis of KD. 

BCL11B was identified as an important role in T-cell function [34], and its lower expression is associated with adverse clinical outcomes for patients with myelodysplastic syndrome [36]. DUSP2 can control the activity of the transcription activator signal transducer and the activator of transcription 3 in its regulation of TH17 differentiation [37], and can also act as an immune checkpoint in T-cell antitumor immunity [38]. DDX24 acts as a regulator of p300 and modulates p53’s control of cellular growth [39]. Previously, Chen et al. [23] reported 3096/3193 CpG (97%) methylation regions with a methylation difference ≥20% between KD and controls, and also revealed hypomethylation. While hypomethylation indicated increased gene expression, almost all the genes were shown to be in an activated status during the acute stage of KD before IVIG treatment, thus suggesting that KD is an acute inflammatory disease. Our noteworthy findings on the suppressed genes found in this study (BCL11B, DUSP2, DDX24, and CDR2) may play a very important role in the balance or regulation of the immune response of KD, such as regulatory T in Th1 and Th2 immune responses. The detailed functions of these genes in KD require further investigation in the future. 

## 5. Conclusions

Activated gene expression represented a majority of the changes seen in the immune response of KD. Nevertheless, insight into suppressed genes in KD would still help us to better understand the pathophysiology of KD. In this study, we first identified CDR2 as a novel suppressed gene for Kawasaki disease via human transcriptome array analysis, and found DDX24 to be associated with CAL formation—both of which may contribute to further understanding of CAL pathogenesis in KD. However, further functional research on these genes is still warranted in the future.

## Figures and Tables

**Table 1:** Basal characteristics of patients with KD and controls.

Characteristic	Healthy Controls (*n* = 19)	Febrile Controls (*n* = 20)	Kawasaki Disease (*n* = 31)	*p*-Value
Male gender, *n* (%)	11 (58%)	15 (75%)	20 (65%)	0.522
Age (y)	3.2 ± 0.4 ^a^	3.5 ± 0.5 ^a^	1.7 ± 0.2 ^b^	<0.001
Age range (y)	1–6	0–8	0–5	
White blood cell (1000/µL)	9.3 ± 0.5 ^a^	7.5 ± 0.9 ^a^	11.4 ± 0.6 ^b^	<0.001
Hemoglobin (g/dL)	12.5 ± 0.2 ^a^	12.2 ± 0.2 ^a^	11.3 ± 0.2 ^b^	<0.001
CRP (mg/L)		18.2 ± 6.0	58.2 ± 11.1	0.003
CAL formation (%)			14 (45.2%)	
IVIG resistance (%)			1 (3.2%)	

CAL, coronary artery lesion; KD, Kawasaki disease; IVIG, intravenous immunoglobulin. Different letters indicate significant differences data expressed as mean ± SEM.

## Data Availability

The datasets generated and analyzed during the current study are not publicly available due to strict ethical regulation of information privacy, but are available from the corresponding author Ho-Chang Kuo upon reasonable request.
